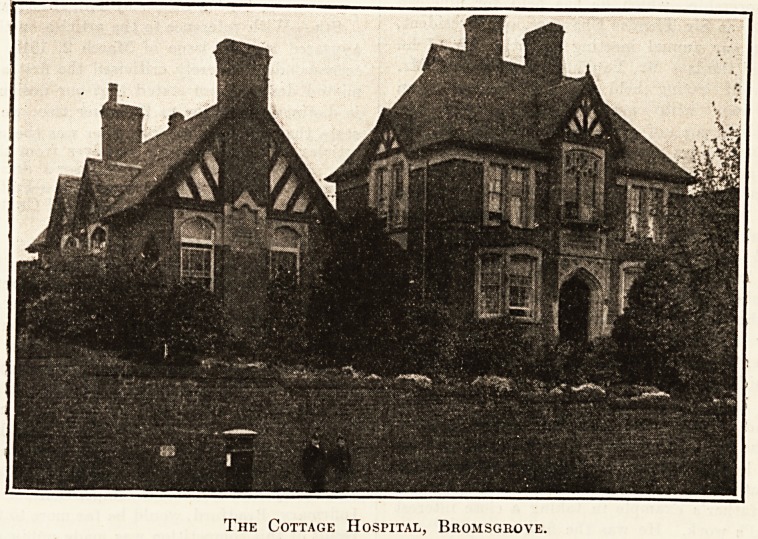# The Cottage Hospital in Fact

**Published:** 1912-03-09

**Authors:** 


### THE COTTAGE HOSPITAL IN FACT.

### The Management and Administration of a Typical Institution.

The Bromsgrove Cottage Hospital, the secretary of which, Mr. J. Clifford Nicholls, courteously gave an interview to our commissioner the other day, is an institution over thirty years old and with thirteen beds: a type, in short, of the firmly-established moderately-sized cottage hospital. The honorary secretary, Mr. Nicholls, and the matron, Miss Polson, very kindly answered a series of questions as to the methods of administration and procedure. Speaking on this subject the Secretary said:

"We have a general Committee of Management, consisting of local persons interested in the hospital, which meets once every month. In addition there is what we call the G.P.C., the General Purposes Committee, virtually a sub-committee of the former, which may meet at any time. In case of complaint on the part of any patient, or of any irregularity that may come to the notice of the matron, the General Purposes Committee, one or other member of which is generally to be found in the hospital during the day, can be instantly summoned. The procedure then is for this committee to go into the matter, and then to prepare a report which it will lay before the general committee at their next meeting. Should, however, the matter in question be exceptionally urgent, at a hint from the General Purposes Committee the Committee of Management could be summoned at very short notice."

"Then as regards the medical staff?"

"The first point to notice is that we have no resident medical officer. Our medical staff consists of six local practitioners, one of whom every twelve months is chosen as the medical officer in charge. The way in which the duties of the medical staff are allocated is as follows: When a patient arrives at the hospital he is at once asked who is his medical practitioner in the town. If he says that he has no particular medical attendant, the medical officer in charge at the time is sent for, and thereupon takes charge of the case. In instances where the patient gives the name of his customary doctor, that gentleman is notified, with the general result that each member of the medical staff looks after his own patients in the hospital. The effect of this is that one or more of the medical staff is found in the hospital every day, and each member of the staff has an equal opportunity of attending those patients who have not already decided upon their own medical man."

"Then how about the dispensing of drugs?"

"We have here only a diminutive dispensary, just for the making up of a few necessary lotions and so on. The dispensing in general is carried out by the three chemists in the town, each of whom is appointed in turn for twelve months to make up the prescriptions. I should add here that we have no out-patient department, and that the hospital is designed for all suitable cases within a radius of five miles of Bromsgrove, except that accidents are always admitted."

"And as regards the other tradesmen?"

"The same arrangement applies. The butchers and the bakers take it in turn to supply the hospital with their goods for a fixed period. We do not have, therefore, in the strict sense, a contract system. It has been the practice of the hospital to rely almost entirely on local tradespeople, partly out of a natural local patriotism and partly as a return for the keen interest which the town takes in the hospital's affairs."

The Cottage Hospital, Bromsgrove.

"What visiting is done?"

"There are at present," said the matron, in reply to this question, "two lady visitors. I have always found that it makes matters easier for the lady visitors if they are sure of a welcome when they arrive, and also that it causes less disturbance to the patients. The result is that they generally informally let me know of the time when they may be expected, usually in the afternoon."

Turning again to the Secretary, he was asked if he had any other point to emphasise in connection with the management. He thereupon referred to rule 2. "This," he said, "provides that the hospital shall be under the management of a committee, consisting of the President, Vice-Presidents, two lady visitors, the medical and dental officers of the hospital, honorary treasurer, honorary secretary, and eleven subscribers who shall be elected at the annual meeting. You will note that the eight of the elected members of the committee who during the preceding year shall have attended the greatest number of meetings shall be re-elected, the remaining three shall retire, and shall not for one year be eligible for re-election. You see the effect of this is that three members of the committee anyway retire each year. That ensures a little new blood to the committee, and though this rule is not very popular among the rejected three, who, often for no fault of their own, through illness it may be, have been unable to attend regularly, yet it is felt that the harshness of the rule's incidence in isolated cases is a small price to pay for the assured fresh element in the committee itself. Incidentally I may add the action of rule 2 lends an additional interest to our annual meeting, for there is no doubt that a position on the hospital's committee is a privilege sought after by our supporters in the town."

"Personal service here is well understood?"

"When we come to the accounts you will see that. In the meantime, as a typical instance of the high services on which the hospital's success has been built, I can instance Mr. John Green, who, to everyone's regret, is retiring from the treasurership, which he has held for nineteen years. In 1892, as Sir Thomas Chavasse, our President, pointed out at our annual meeting recently, Mr. John Green succeeded the late Mr. Laughton as treasurer, Mr. Laughton himself having held the post for seventeen years, and was, with my father, the late Mr. George Nicholls, one of the prime-movers of the institution. Mr. John Green, who before 1892 had been co-secretary with the late Mr. Nash Creswell, another benefactor, was acknowledged by Sir Thomas Chavasse and other authorities to be a most successful hospital financier. Indeed, the accounts show this. In 1892, the first year in which Mr. Green was treasurer, the hospital's income was £491—this year, as my nearly finished report will show, it is £835. The expenditure at these same dates has been £410 and £745. Thus not only has our expenditure doubled, but our income after twenty years still shows a substantial balance on the right side. A permanent memorial is to be presented to the hospital to commemorate the services of Mr. Green, and will take the form of a framed portrait. Mr. Horton has kindly promised to succeed him as treasurer, and his legal knowledge and experience are bound to be of great value to the committee. I am only following my father's example in taking a close interest in this hospital's work. He was the first secretary."

## Figures and Tables

**Figure f1:**